# Early exposure to thirdhand cigarette smoke affects body mass and the development of immunity in mice

**DOI:** 10.1038/srep41915

**Published:** 2017-02-03

**Authors:** Bo Hang, Antoine M. Snijders, Yurong Huang, Suzaynn F. Schick, Pin Wang, Yankai Xia, Christopher Havel, Peyton Jacob, Neal Benowitz, Hugo Destaillats, Lara A. Gundel, Jian-Hua Mao

**Affiliations:** 1 Biological Systems and Engineering Division, Lawrence Berkeley National Laboratory, Berkeley, CA 94720, USA; 2 Department of Medicine, Division of Occupational and Environmental Medicine, University of California, San Francisco, Box 0843, San Francisco, CA 94143, USA; 3 Department of Gastroenterology, The Affiliated Drum Tower, Clinical Medical School, Nanjing Medical University, Nanjing, Jiangsu 210008, China; 4 State Key Laboratory of Reproductive Medicine, Institute of Toxicology, Nanjing Medical University, Nanjing 211166, China; 5 Indoor Environment Group, Lawrence Berkeley National Laboratory, Berkeley, CA 94720, USA

## Abstract

Thirdhand smoke (THS) is the fraction of cigarette smoke that persists in indoor environments after smoking. We investigated the effects of neonatal and adult THS exposure on bodyweight and blood cell populations in C57BL/6 J mice. At the end of neonatal exposure, THS-treated male and female mice had significantly lower bodyweight than their respective control mice. However, five weeks after neonatal exposure ended, THS-treated mice weighed the same as controls. In contrast, adult THS exposure did not change bodyweight of mice. On the other hand, both neonatal and adult THS exposure had profound effects on the hematopoietic system. Fourteen weeks after neonatal THS exposure ended, eosinophil number and platelet volume were significantly higher, while hematocrit, mean cell volume, and platelet counts were significantly lower compared to control. Similarly, adult THS exposure also decreased platelet counts and increased neutrophil counts. Moreover, both neonatal and adult THS exposure caused a significant increase in percentage of B-cells and significantly decreased percentage of myeloid cells. Our results demonstrate that neonatal THS exposure decreases bodyweight and that THS exposure induces persistent changes in the hematopoietic system independent of age at exposure. These results also suggest that THS exposure may have adverse effects on human health.

In recent years, potential health concerns have been raised about thirdhand smoke (THS), a much less understood type of cigarette smoke exposure. THS is residual tobacco smoke that clings to indoor surfaces, and re-emission of gases and resuspension of particles from contaminated surface materials after active smoking has ceased^1^. THS also includes novel chemical compounds generated *de novo*, as demonstrated by the recent studies showing that surface-bound nicotine, a major constituent of THS, reacts with nitrous acid (HONO) to form carcinogenic tobacco-specific nitrosamines (TSNAs)^2^, and with ozone (O_3_) to yield oxidants^3^,^4^ and ultrafine asthmagenic particles^5^. While gas phase concentrations decrease over time due to ventilation, the indoor residence time of surface-bound semivolatile and non-volatile THS constituents can be very long (weeks to months), and their chemical transformations may render these compounds more harmful over time.

The toxicological and adverse health impacts of active smoking and secondhand smoke (SHS) have been extensively studied before. However, the process of characterizing the potential biological and health effects of THS is just beginning. Chemical analyses of THS composition reveal that THS contains many classes of toxic compounds, including both semi-volatile (SVOCs) and volatile organic compounds (VOCs), as well as chemicals with incomplete toxicity testing^2^,^3^,^4^,^5^,^6^,^7^. Therefore, THS may contain many toxicants similar to those in mainstream or SHS and new toxic compounds generated from chemical transformations.

Oxidants in cigarette smoke can cause local and systemic inflammation. Recently, Hang *et al*. demonstrated for the first time that exposure to laboratory-generated THS causes significant DNA damage in human cell lines^8^. Bahl *et al*. showed cytotoxicity of THS in both mouse and human cell lines^9^. Martins-Green *et al*. reported animal studies showing that THS exposure resulted in damage to multiple organs and behavioral alterations in mice^10^. Karim *et al*. showed that THS exposure increases the risk of thrombosis-based disease states^11^. Xu *et al*. found that the exposure to THS at very low concentrations caused distinct metabolic changes in two different types of male reproductive cell lines^12^. These studies and others suggest that THS can cause adverse health effects that need further investigation.

Small children are a particularly vulnerable population who are exposed to THS toxicants through inhalation, ingestion and dermal contact. By analyzing nicotine and nitrosamines/TSNAs in house samples, Ramirez *et al*. found that the calculated cancer risk for children (1 to 6 years old) is increased^13^. Although these results suggest that THS is a potential health threat to the infants and young children who are in smokers’ homes, virtually nothing is known about the specific health effects of THS exposure in infants and children.

The link between SHS exposure and body weight and immunological parameters has been investigated extensively in the past^14^,^15^,^16^,^17^. In this study, we have used an animal model to extend these observations by investigating the effect of THS exposure on bodyweight and the hematopoietic system in mice during two specific life stages: neonatal (from birth until weaning) and early adulthood (from 12 to 15 weeks of age).

## Results

### Neonatal THS exposure significantly reduces bodyweight

**Figure 1:**
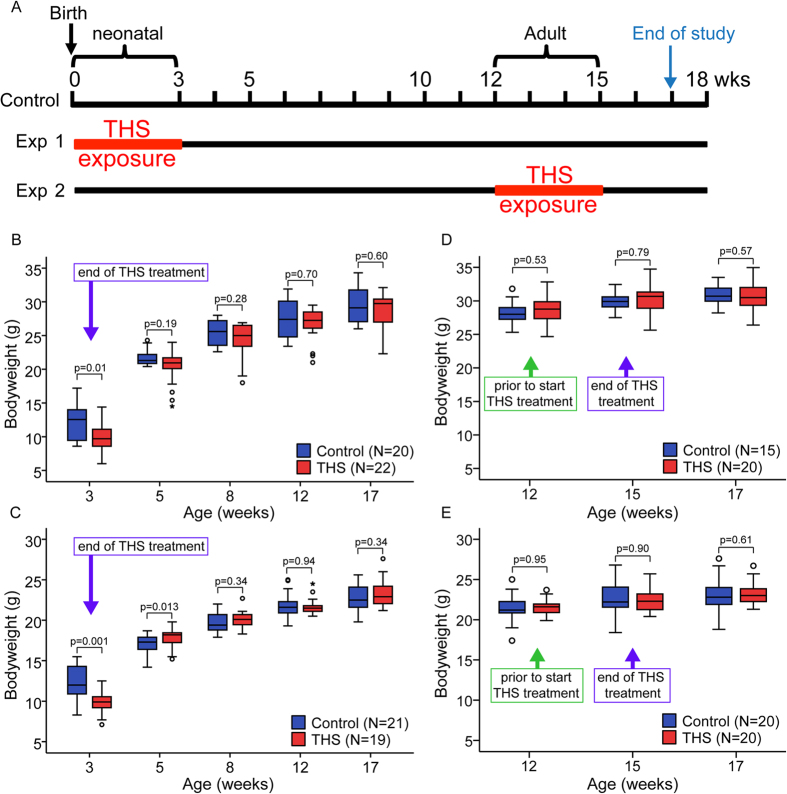
Neonatal THS exposure significantly reduces bodyweight. **(A**) Schematic depicting overall experimental strategy. Mice were exposed to THS from birth to 3 weeks of age (neonatal exposure group) or from 12 to 15 weeks of age (adult exposure group). Bodyweight was measured at specific timepoints and the composition of the hematopoietic system was measured at 17 weeks of age. (**B,C**) Effect of neonatal THS exposure on bodyweight of (**B**) male and (**C**) female mice. (**D,E**) Effect of adult THS exposure on bodyweight of (**D**) male and (**E**) female mice before and after exposure to THS. The boxplots indicate the 25^th^ (bottom) and 75^th^ percentile (top) and the dark line in the middle of the box is the median. The whiskers extend 1.5 times the interquartile range. Outliers are indicated with open circles and extreme values (more than three times interquartile range) are indicated with an asterisk. The p-values were obtained from non-parametric (Mann-Whitney) test.

To examine the effects of THS exposure on health, we treated C57BL/6 J mice by placing 5 × 5 cm^2^ swatches of THS-exposed 100% cotton terry cloth in their cages for 3 weeks. THS exposure cages contained standard bedding material plus THS cloth, while control cages contained standard bedding only (Fig. 1A). The cloth swatches were changed once a week. To study potential age-dependent effects of THS exposure, two cohorts of mice were subjected to THS exposure: one exposed from birth to 3 weeks, and the other exposed from 12–15 weeks of age (Fig. 1A). For the neonatal exposure cohort, both the THS and control groups contained 6 litters of mice. The distribution of litter sizes in THS and control groups (average 7 mice per litter) was identical to avoid the confounding effect of litter size on bodyweight (Table S1). After weaning, all pups were separated by sex and housed under standard conditions. As shown in Fig. 1B,C, at weaning THS-exposed male and female mice had significantly lower bodyweight than control mice (p < 0.01). However, after weaning, and without further exposure to THS, the THS-exposed male and female mice gained significantly more bodyweight to catch up to the control mice at 5 weeks. The THS-exposed mice maintained normal body mass at 12 and 17 weeks (Fig. 1B,C). Interestingly, at 5 weeks, although no difference in bodyweight was observed between treated and control male mice (Fig. 1B), the THS-treated female mice weighed more than the controls (Fig. 1C). In contrast, THS exposure during adulthood had no effect on bodyweight in male or female mice (Fig. 1D,E). Putting it all together, we conclude that THS exposure can reduce mice bodyweight in an age-dependent manner.

### THS exposure significantly and persistently affects the abundance of cell populations in blood

**Figure 2:**
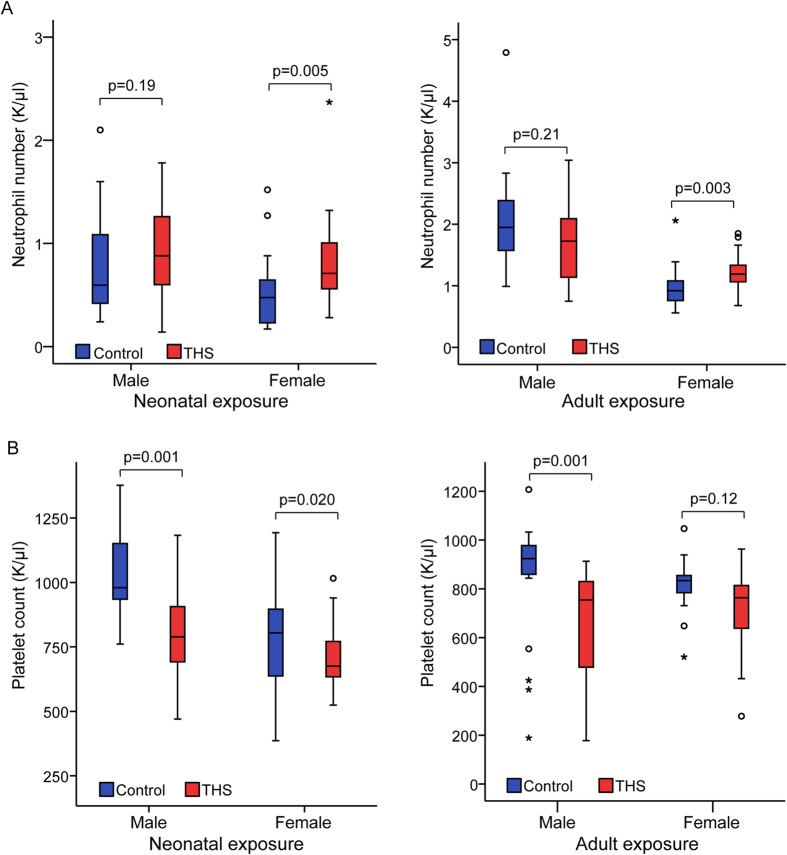
THS exposure causes persistent alterations in the hematopoietic system. (**A**) THS exposure significantly increases neutrophil number (K/μl) in female mice, but not in male mice. (**B**) Platelet counts (K/μl) are significantly decreased in neonatally exposed THS male and female mice and adult exposed male mice. The boxplots indicate the 25^th^ (bottom) and 75^th^ percentile (top) and the dark line in the middle of the box is the median. The whiskers extend 1.5 times the interquartile range. Outliers are indicated with open circles and extreme values (more than three times interquartile range) are indicated with an asterisk. The p-values were obtained from non-parametric (Mann-Whitney) test.

The complete blood count (CBC) is a standard test to assess general health status. Therefore, we used a 20-parameter CBC to investigate the effects of THS exposure on the abundance of different cell populations in mouse blood. When tested at 17 weeks, both male and female mice in the neonatal THS exposure cohort had almost 3 times as many eosinophils (EO) and correspondingly higher EO percentages than controls (Table 1). Some sex-dependent changes in the neonatal exposure group were also observed, including significantly increased neutrophil (NE) number and percent (Fig. 2A, left panel) in THS-treated females and increased basophil (BA) number and percent in treated males. A similar increase in neutrophil number was observed in female mice exposed during adulthood (Fig. 2A, right panel, Table S2), suggesting that some THS exposure effects on the hematopoietic system are independent of age at the time of exposure.

Apparently, red blood cells (RBC) were not affected, as both RBC counts and hemoglobin (HB) levels were not significantly altered. However, we found that platelet counts were significantly lower in neonatal-exposed male and female mice and adult-exposed male mice (Fig. 2B, Table 1, Table S2). Additionally, hematocrit, mean cell volume, and red cell distribution width were significantly lower in neonatal-exposed male and female mice and mean platelet volume was significantly higher (Table 1). In adult THS-exposed mice, we also observed a significant decrease in mean cell volume and red cell distribution width in female mice only (Table S2). Taken together, we concluded that neonatal THS exposure caused persistent changes in hematopoiesis and that THS exposure can change certain hematopoietic parameters independent of age at exposure.

### THS exposure persistently affects the abundance of B-cells and myeloid cells

**Figure 3:**
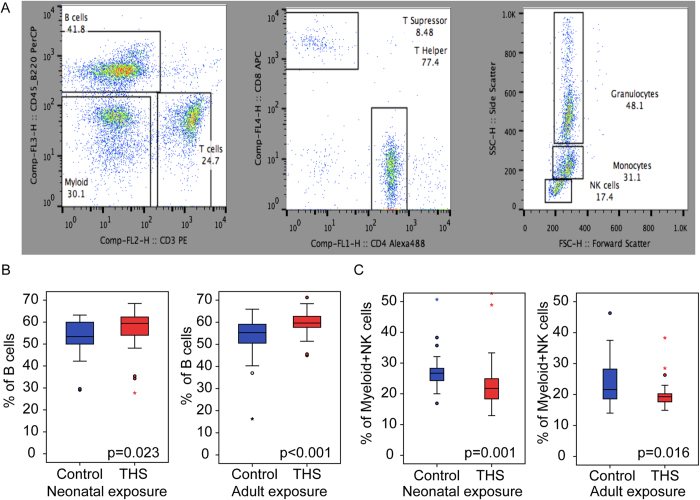
THS exposure alters lymphoblastic subpopulations. (**A**) Representative FACS dot plots for analyzing flow cytometry data by FlowJo. White blood cells were separated into CD3+ T-cells, CD45.B220+ B-cells and CD3−/CD45.B220− myeloid cell fractions (left). CD3+ T-cells were further separated into CD3+/CD4+ T-helper cells and CD3+/CD8+ T-suppressor cells. The myeloid/NK fraction was separated using forward and side scatter into granulocyte, monocyte and NK enriched fractions. (**B**) THS exposure significantly increases percentage of B-cells in neonatally exposed (left) and adult exposed (right) mice. (**C**) THS exposure significant decreases percentage of myeloid/NK fraction (CD3−/CD45.B220−) in neonatally exposed (left) and adult exposed (right) mice. The boxplots indicate the 25^th^ (bottom) and 75^th^ percentile (top) and the dark line in the middle of the box is the median. The whiskers extend 1.5 times the interquartile range. Outliers are indicated with open circles and extreme values (more than three times interquartile range) are indicated with an asterisk. The p-values were obtained from non-parametric (Mann-Whitney) test.

Although there was no difference in absolute blood lymphocyte numbers between THS-exposed and control mice, further analysis by FACS (fluorescence-activated cell sorting) revealed significant changes in lymphocyte subpopulations. Figure 3A shows representative FACS dot plots analyzed by FlowJo™, including T- (CD3+/CD45.B220−), B- (CD3−/CD45.B220+) and myeloid/NK cell fractions (CD3−/CD45.B220−), T-suppressor (CD3+ and CD4−/CD8+) and T-helper (CD3+ and CD4+/CD8−) cells and the NK, monocyte and granulocyte enriched fractions (CD3−/CD45.B220− and FSC and SSC). We observed that both neonatal and adult THS exposure resulted in a significant increase in the B-cell fraction (Fig. 3B, Tables S3 and S4) accompanied by a significant decrease in the myeloid/NK cell fraction (Fig. 3B, Tables S3 and S4). Since the NK enriched fractions were not significantly different between THS and control samples (Tables S3 and S4), we attributed this decrease to a change in the myeloid fraction (granulocytes, erythrocytes and platelets) consistent with our CBC data (Tables 1 and S2). While the T-cell fraction remained unchanged (Tables S3 and S4), the percentage of T-suppressor cells within the T-cell fraction was significantly increased after neonatal THS exposure only (Table S3).

## Discussion

Because of children’s exploratory behavior and metabolic properties, it is likely that they are at increased health risk when exposed to THS toxicants. For example, children who spend a lot of time on THS-laden carpets could breathe in a significantly larger amount of dust-bound pollutants than adults, while their skin is in near-constant contact with carpet or other furniture surfaces. Dust ingestion was identified as the dominant (80% of total intake) source of exposure to indoor semivolatile environmental pollutants in toddlers^18^. While THS and SHS exposures often overlap, a recent study at a neonatal intensive care unit showed that infants whose parents smoke had THS markers such as cotinine, hydroxycotinine and NNAL in their urine, illustrating that THS is present even in environments where active smoking is absent and extreme hygienic precautions are taken^19^. Although there have been a few studies suggesting that THS is a potential health threat to infants and young children who are in smokers’ homes, only recently is the association between exposure to THS and specific biological or health impacts being explored.

The most striking finding of this study was a significant reduction in weight gain in both THS-treated male and female mice exposed to THS from birth until weaning at 3 weeks of age. Similarly exposed adult mice did not show THS exposure induced reduction in bodyweight. After the neonatal exposure to THS ended, the bodyweight of exposed mice caught up to control mice at 5, 8, 12 and 17 weeks. To our best knowledge, this is the first report of bodyweight change caused by exposure to THS under conditions that mimic human exposure. The links between active cigarette smoking, smoking cessation and bodyweight change have been investigated extensively in the past. In some previous studies it has been shown that adult smokers lose weight as compared to non-smokers, and tend to gain weight after they quit smoking^20^. It is less clear whether smoking during adolescence leads to significant bodyweight loss, although many people consider smoking as a way to control weight. Our data are in agreement with such observations.

Although the exact mechanisms underlying the changes in weight gain we observed are still unclear, tobacco use is known to be associated with appetite suppression and nicotine is considered an appetite suppressant. Moreover, nicotine has been reported to induce bodyweight loss in both human and rodent studies through mechanisms including affecting hormones that control caloric intake and fat metabolisms^20^,^21^,^22^,^23^,^24^. Nicotine is the main constituent in THS, as identified in various experimental and field samples^7^,^25^. Alternative explanations for the observed reduction in weight gain of THS-exposed pups include an alteration in nursing behavior or a change in the amount of milk available for suckling. Future studies will need to be conducted to clarify this.

There is a link between active and passive smoking and changes in complete blood counts, which may underlie the pathophysiological mechanism of the biological effects of tobacco smoking^26^,^27^,^28^. Most of the statistically significant differences in immunological parameters presented in neonatal THS-exposed mice also have clinical relevance. For example, since increased numbers of eosinophils are associated with a variety of disorders including parasitic infections and allergic diseases, the increased number and percentage of both eosinophils in both sexes and the increase in basophils in male mice suggests that THS exposure may have effects on allergy and atopy. Increased neutrophils observed in neonatal and adult exposed female mice, suggest increases in acute inflammation, intoxication or tissue damage may be caused by THS toxicants. Decreased platelets levels, also observed in adult exposed male mice, might have been associated with platelet dyspoiesis and/or platelet destruction or over-consumption. Increased mean platelet volume indicates that bone marrow compensation in platelet generation remained unaffected in these mice and suggests that the lower number of platelets observed may be due to increased platelet destruction. We also observed that certain changes in blood cell counts are dependent on sex possibly due to baseline physiological variations between male and female mice.

We further studied the effect of neonatal exposure to THS on specific immune subpopulations in mouse blood. As shown in Fig. 3, both neonatal and adult THS exposure resulted in a significant increase in percentage of B cells, accompanied by significantly decreased myeloid cell numbers. Increased B cells may reflect an increased allergic/immune response to THS chemicals adsorbed into the body, whereas a decrease in blood myeloid cells may reflect a certain degree of bone marrow depression. It should be noted that the changes observed in both the blood cell counts and lymphocyte numbers were still seen 14 weeks after exposure cessation in the neonatal cohort and 2 weeks after exposure cessation in the adult cohort. These timepoints were chosen to be able to compare the blood analyses of the adult and neonatal THS cohorts without age being a confounding factor. Future time course studies can be conducted to investigate the time-dependent changes in blood count differences. Our study did not include repeated blood collections so as to avoid disturbing the immune status of individual mice. We have not associated the observed changes in weight gain or blood cell populations caused by THS exposure with health outcomes in mice. However, the existing literature suggests that such changes could contribute to adverse health effects. Previous studies demonstrating health effects of THS exposure used the same strain of mice (C57BL/6), but employed longer exposures (24 weeks), and a method in which the entire cage and all bedding were exposed to THS^10^.

In conclusion, our data show that THS exposure can inhibit normal weight gain in neonatal mice. We have also shown that THS exposure, during infancy and during early adulthood, changes the circulating populations of blood cells from both the myeloid and lymphoid lineages. Our research also shows that neonates are more vulnerable to potential health effects caused by THS exposure than adults. Our findings provide further evidence that THS exposure can have biological effects and supports nonsmoking policies as the best way to protect nonsmokers from the health effects of tobacco smoke exposure.

## Methods

### Generation of THS samples

The THS samples were generated in a laboratory system to simulate chronic THS exposure, as described in the previous study^8^. Briefly, 100% cotton terrycloth samples were repeatedly exposed to cigarette smoke in a 6-m^3^ stainless steel chamber for a total of 550 hours over 1,190 days. The smoke exposure chamber was ventilated for approximately 550 hours during this time. During smoking, a total of 5,600 mg of particulate material was introduced into the steel chamber. This is equivalent to the smoke from 400–700 cigarettes over 3 years and 4 months, or approximately 1/3–2/3 of a cigarette per day.

### Animal exposure and bodyweight studies

C57BL/6 mice were divided into experimental and control groups. The neonatal experimental group was exposed to THS from birth for 3 weeks; the control group was never exposed to THS and was housed separately from the THS-exposed mice. The adult experimental group was exposed to THS from 12 to 15 weeks of age. All mice were fed a standard chow diet (percent calories: 58% carbohydrate, 28.5% protein, and 13.5% fat). Mice were bedded on the THS-laden cloths along with standard bedding in mouse cages and then switched to standard bedding only. The cloth swatches were changed once a week with standard cage change. The cloth was the sole source of smoke exposure. Possible routes of THS exposure include inhalation, ingestion and dermal absorption. There was 0.85 g (5 × 5 cm^2^ swatches) of THS-exposed cloth per cage, with a nicotine loading of 20 μg/g. Assuming that uptake of nicotine through ingestion, inhalation and dermal routes was quantitative, the predicted dose was 50 μg/day/kg of bodyweight. This value is comparable to the ingestion exposure of a toddler estimated by Bahl *et al*.^29^. Control animals were housed separately on standard bedding. The mice were weighed at 3, 5, 8, 12 and 17 weeks.

### Ethics Statement

All animal experiments were performed at Lawrence Berkeley National Laboratory and the study was carried out in strict accordance with the Guide for the Care and Use of Laboratory Animals of the National Institutes of Health. The animal use protocol was approved by the Animal Welfare and Research Committee of the Lawrence Berkeley National Laboratory.

### Blood cell analysis

Whole blood was collected by retro-orbital bleeding into EDTA-coated tubes (Fisher Scientific). A complete blood cell count was acquired using a HemaVet950FS and specific lymphocyte subpopulations were assessed by FACS with cell specific markers for B-cells, T-cells, T-helper and T-suppressor cells at 17-weeks using the following antibodies (BD Biosciences): rat anti mouse CD3-PE; rat anti mouse CC45R/B220 PerCP; rat anti mouse CD8a antibody APC; rat anti mouse CD4 antibody Alexa 488. The percentages of cells in blood were determined on BD FACS Calibur (Becton Dickinson) and data were analyzed with FlowJo software (Tree Star, Inc.).

### Statistics analysis

The differences in bodyweight, the cellular components of blood and lymphocyte subpopulations between THS-treated and control group were assessed by Mann-Whitney test. Results with p-value < 0.05 were judged to be significant. Statistical analysis was performed using SPSS version 12.0 (SPSS, Chicago, IL).

## Additional Information

**How to cite this article:** Hang, B. *et al*. Early exposure to thirdhand cigarette smoke affects body mass and the development of immunity in mice. *Sci. Rep.* **7**, 41915; doi: 10.1038/srep41915 (2017).

**Publisher's note:** Springer Nature remains neutral with regard to jurisdictional claims in published maps and institutional affiliations.

## Supplementary Material

Supplementary Tables

## Figures and Tables

**Table 1:** Effect of neonatal THS exposure on the cellular components of blood.

Cellular component	Male			Female		
	Control (N = 20)	THS (N = 21)	p-values^$^	Control (N = 20)	THS (N = 19)	p-values^$^
**WBC number (K/μL)**	7.96 (1.86)^#^	8.84 (2.02)	0.22	7.90 (1.70)	8.21 (2.24)	0.96
**NE number (K/μL)**	0.80 (0.50)	0.95 (0.46)	0.19	0.53 (0.36)	0.84 (0.46)	**0.005**
**LY number (K/μL)**	6.55 (1.91)	7.09 (1.66)	0.59	6.74 (1.44)	6.77 (1.68)	0.63
**MO number (K/μL)**	0.56 (0.23)	0.65 (0.32)	0.38	0.57 (0.22)	0.46 (0.20)	0.14
**EO number (K/μL)**	0.039 (0.076)	0.11 (0.11)	**0.016**	0.039 (0.076)	0.10 (0.13)	**0.023**
**BA number (K/μL)**	0.013 (0.029)	0.041 (0.043)	**0.011**	0.014 (0.023)	0.033 (0.047)	0.51
**NE percent (%)**	10.90 (7.79)	10.81 (5.10)	0.52	6.66 (3.90)	10.03 (3.31)	**0.006**
**LY percent (%)**	81.28 (10.51)	80.40 (6.90)	0.12	85.55 (4.85)	82.98 (4.66)	0.092
**MO percent (%)**	7.21 (3.10)	7.17 (3.15)	0.99	7.18 (2.45)	5.46 (1.33)	0.056
**EO percent (%)**	0.45 (0.81)	1.17 (1.13)	**0.037**	0.43 (0.77)	1.21 (1.31)	**0.016**
**BA percent (%)**	0.16 (0.32)	0.45 (0.45)	**0.007**	0.16 (0.25)	0.38 (0.50)	0.26
**Red blood cell (M/μL)**	8.61 (0.41)	8.05 (1.05)	0.054	8.59 (0.32)	8.41 (0.61)	0.53
**Hemoglobin (g/dL)**	11.42 (1.14)	10.78 (1.53)	0.23	11.38 (1.16)	11.02 (0.93)	0.43
**Hematocrit (%)**	39.62 (2.58)	34.52 (4.27)	**<0.001**	40.13 (2.68)	36.18 (2.24)	**<0.001**
**Mean cell volume (fL)**	46.31 (2.40)	42.96 (1.90)	**<0.001**	46.71 (2.72)	43.08 (1.76)	**<0.001**
**Mean cell hemoglobin (pg)**	13.35 (1.35)	13.40 (0.85)	0.90	13.24 (1.18)	13.15 (1.17)	0.48
**Mean cell hemoglobin concentration (g/dL)**	28.96 (3.60)	31.23 (2.03)	**0.039**	28.50 (3.76)	30.49 (2.34)	0.07
**Red cell distribution width (%)**	17.82 (1.85)	17.01 (0.60)	**0.009**	18.74 (1.63)	16.88 (0.59)	**<0.001**
**Platelet count (K/μL)**	843.65 (252.80)	673.69 (223.56)	**0.001**	819.55 (107.94)	710.66 (171.47)	**0.020**
**Mean platelet volume (fL)**	4.64 (0.20)	4.82 (0.27)	**0.018**	4.70 (0.15)	4.86 (0.24)	**0.027**

^$^p-values were obtained from non-parametric (Mann-Whitney) test, the bold p-values indicate significance; ^#^Mean (STD).

WBC: White blood cell; NE: Neutrophil; LY: Lymphocyte; MO: Monocyte; EO: Eosinophil; BA: Basophil; K/μL: x1000/μL.
