# Supplementary material for: Early exposure to thirdhand cigarette smoke affects body mass and the development of immunity in mice
Source: Sci Rep. 2017 Feb 3;7:41915. doi: 10.1038/srep41915 (PMC5291208; doi:10.1038/srep41915)
Supplement: Supplementary Tables [file srep41915-s1.pdf]

Early exposure to thirdhand cigarette smoke affects body mass and the development of immunity in mice

Bo Hang[1], Antoine M Snijders[1], Yurong Huang[1], Suzaynn F Schick[2], Pin Wang[3], Yankai Xia[4], Christopher Havel[2], Peyton Jacob III[2], Neal Benowitz[2], Hugo Destaillats[5], Lara A Gundel[5], and Jian-Hua Mao[1*]

Table S1. Distribution of litter sizes in THS and control groups

Experimental group	THS	Control
Litter size	5	5
	5	5
	6	6
	7	7
	9	9
	9	9

Table S2. Effect of adult exposure to THS on the cellular components of blood.

	Male			Female		
	Control (N=15)	THS (N=20)	p-values$	Control (N=20)	THS (N=20)	p-values$
White blood cell number (WBC) (K/µL)	7.99 (2.28)*	8.80 (2.82)	0.58	6.27 (2.11)	7.92 (1.68)	0.003
Neutrophil number (NE) (K/µL)	2.09 (0.92)	1.71 (0.67)	0.21	0.97 (0.34)	1.24 (0.30)	0.003
Lymphocyte number (LY)(K/µL)	5.74 (2.09)	6.84 (2.17)	0.17	5.10 (1.78)	6.55 (1.42)	0.003
Monocyte number (MO) (K/µL)	0.13 (0.04)	0.16 (0.08)	0.35	0.13 (0.06)	0.10 (0.05)	0.083
Eosinophil number (EO) (K/µL)	0.03 (0.03)	0.07 (0.09)	0.10	0.05 (0.07)	0.02 (0.03)	0.14
Basophil (BA) (K/µL)	0.01 (0.01)	0.03 (0.04)	0.01	0.01 (0.02)	0.01 (0.01)	0.28
NE percent (%)	27.54 (13.46)	19.33 (5.04)	0.03	16.44 (5.86)	15.69 (2.57)	0.61
LY percent(%)	70.27 (13.66)	77.89 (5.34)	0.04	79.59 (6.96)	82.62 (2.30)	0.39
MO percent (%)	1.74 (0.53)	1.79 (0.67)	0.78	2.10 (0.78)	1.32 (0.59)	0.001
EO percent (%)	0.36 (0.41)	0.70 (0.68)	0.14	0.68 (0.78)	0.28 (0.29)	0.074
BA percent (%)	0.10 (0.14)	0.28 (0.30)	0.02	0.19 (0.25)	0.09 (0.13)	0.051
Red blood cell (RBC) (M/µL)	8.77 (0.32)	8.60 (0.57)	0.31	8.93 (0.40)	8.75 (0.25)	0.10
Hemoglobin (Hb) (g/dL)	12.10 (0.48)	11.43 (0.96)	0.01	12.23 (0.58)	12.18 (0.29)	0.64
Hematocrit (HCT) (%)	39.80 (1.50)	39.36 (2.48)	0.69	41.02 (1.93)	39.98 (1.22)	0.025
Mean cell volume (MCV) (fL)	45.40 (0.72)	45.80 (0.80)	0.14	46.17 (1.06)	45.71 (0.34)	0.030
Mean cell hemoglobin (MCH) (pg)	13.82 (0.38)	13.29 (0.50)	0.001	13.77 (0.32)	13.93 (0.25)	0.25
Mean cell hemoglobin concentration (MCHC) (g/dL)	30.41 (0.64)	29.02 (1.14)	0.001	29.82 (0.88)	30.48 (0.59)	0.007
Red cell distribution width (RDW) (%)	17.28 (0.57)	17.04 (0.63)	0.15	17.18 (0.82)	16.51 (0.41)	0.008
Platelet count (PLT) (K/µL)	1030.27 (178.14)	811.30 (159.58)	0.001	782.60 (208.61)	710.15 (127.13)	0.12
Mean platelet volume (MPV) (fL)	4.45 (0.15)	4.43 (0.15)	0.75	4.57 (0.21)	4.47 (0.11)	0.098

$p-values were obtained from Mann-Whitney test; #Mean (STD).

Table S3. Effect of neonatal exposure to THS on the cellular components of lymphocytes

Cell type	Control (N=25)	THS (N=40)	p-value#
% B Cells	53.06 (8.96)*	57.08 (8.65)	0.023
% Myeloid+NK cells	28.62 (9.06)	23.87 (10.10)	0.001
% Granulocytes / Myeloid cells	45.11 (11.46)	41.59 (11.87)	0.22
% Monocytes / Myeloid cells	28.57 (7.30)	28.73 (5.54)	0.81
% NK cells	19.83 (5.77)	19.61 (5.50)	0.84
% T cells	14.79 (3.24)	15.49 (4.07)	0.61
% T CD4&CD8 double negative cells	6.76 (2.15)	6.63 (1.41)	0.73
% T helper / T cells	58.27 (5.55)	59.02 (3.82)	0.79
% T suppress / T cells	27.28 (7.12)	30.16 (5.02)	0.046

#p-values were obtained from Mann-Whitney test; *Mean (STD).

Table S4. Effect of adult exposure to THS on the cellular components of lymphocytes

Cell type	Control (N=35)	THS (N=40)	p-value[#]
% B Cells	53.51 (9.09)*	59.74 (4.97)	<0.001
% Myeloid+NK cells	24.79 (10.97)	19.74 (4.02)	0.016
% Granulocytes / Myeloid cells	24.66 (5.58)	26.07 (4.24)	0.11
% Monocytes / Myeloid cells	24.66 (5.58)	19.74 (4.02)	0.093
% NK cells	35.21 (10.83)	36.46 (6.21)	0.81
% T cells	19.21 (5.06)	18.20 (3.88)	0.33
% T CD4&CD8 double negative cells	9.72 (8.51)	8.58 (3.57)	0.74
% T helper / T cells	52.21 (7.42)	53.70 (5.05)	0.49
% T suppress / T cells	37.15 (3.59)	36.42 (3.63)	0.18

[#]p-values were obtained from Mann-Whitney test; [*]Mean (STD).
